# Differential expression of *TLR* and *CXCR* genes in mammary HC11 cells challenged with *Bacillus cereus* and *Bacillus subtilis*: Implications for mastitis resistance

**DOI:** 10.14202/vetworld.2025.1014-1024

**Published:** 2025-04-25

**Authors:** Nova Dilla Yanthi, Anneke Anggraeni, Syahruddin Said, Sugiyono Saputra, Retno Damayanti Soejoedono, Muladno Muladno, Nina Herlina, Ima Fauziah, Herjuno Ari Nugroho, Mukh Fajar Nasrulloh, Rida Tiffarent

**Affiliations:** 1 Research Center for Applied Microbiology, Research Organization for Life Sciences and Environment, National Research and Innovation Agency, Cibinong Science Center, Jl. Raya Jakarta - Bogor KM. 46, Cibinong, Bogor 16911, West Java, Indonesia; 2 Research Center for Animal Husbandry, Research Organization for Agriculture and Food, National Research and Innovation Agency, Cibinong Science Center, Jl. Raya Jakarta - Bogor KM. 46, Cibinong, Bogor 16911, West Java, Indonesia; 3 Research Center for Applied Zoology, Research Organization for Life Sciences and Environment, National Research and Innovation Agency, Cibinong Science Center, Jl. Raya Jakarta - Bogor KM. 46, Cibinong, Bogor 16911, West Java, Indonesia; 4 Division of Medical Microbiology, School of Veterinary Medicine and Biomedical Sciences, Bogor Agricultural University (IPB University), Jl. Agatis, Kampus IPB Dramaga, Bogor 16680, West Java, Indonesia; 5 Department of Animal Production and Technology, Faculty of Animal Science, Bogor Agricultural University (IPB University), Jl. Agatis, Kampus IPB Dramaga, Bogor 16680, West Java, Indonesia; 6 Research Centre for Veterinary Science, Research Organization for Health, National Research and Innovation Agency, Cibinong Science Center, Jl. Raya Jakarta - Bogor KM. 46, Cibinong, Bogor 16911, West Java, Indonesia

**Keywords:** *Bacillus cereus*, *Bacillus subtilis*, chemokine receptors, gene expression, HC11 cells, innate immunity, mastitis, toll-like receptors

## Abstract

**Background and Aim:**

Mastitis remains a major health challenge in dairy cattle, often caused by Gram-positive pathogens. Toll-like receptors (TLRs) and chemokine receptors (CXCRs) play essential roles in the innate immune response of mammary epithelial cells (MECs). However, the differential expression of these genes in response to specific mastitis-causing *Bacillus* spp. has not been comprehensively evaluated. This study aimed to characterize the temporal gene expression patterns of TLR and CXCR family members in murine mammary epithelial HC11 cells exposed to *Bacillus cereus* and *Bacillus subtilis*, thereby providing insights into their immunological roles in mastitis pathogenesis.

**Materials and Methods:**

HC11 cells were cultured and infected with *B. cereus* and *B. subtilis* (5 × 10^7^ colony-forming units/mL) and incubated at 37°C with 95% O_2_ and 5% CO_2_ for 48 h in RPMI 1640 medium supplemented with serum and antibiotics. Gene expression of interleukin (IL)-6, IL-8, TLR2, TLR4, IL-1 alpha (IL-1α), and CXCR1 was evaluated by quantitative real-time polymerase chain reaction at 0, 6, 12, 24, 48, and 72 h post-infection. Expression levels were normalized to glyceraldehyde-3-phosphate dehydrogenase and analyzed using ΔCt methods and Spearman correlation.

**Results:**

TLR2 exhibited a biphasic expression pattern, with early upregulation followed by suppression, while TLR4 showed higher expression in response to *B. subtilis* than *B. cereus*. IL-6 displayed prolonged expression under *B. subtilis* challenge but was transient under *B. cereus* exposure. IL-1α showed consistent expression across both bacterial challenges, suggesting its potential as a stable biomarker for mastitis susceptibility. CXCR1 exhibited delayed but sustained expression, indicative of its role in secondary neutrophil recruitment. IL-8 expression correlated with early immune activation and chemotactic signaling.

**Conclusion:**

The immune response of HC11 MECs to Gram-positive bacterial infection is gene- and pathogen-specific. *TLR* and *CXCR* genes show distinct temporal profiles, underscoring their utility in understanding epithelial-driven immune defense. These findings provide molecular insights into mastitis pathogenesis and identify IL-1α, IL-6, and CXCR1 as promising targets for genetic selection and therapeutic intervention.

## INTRODUCTION

Mastitis is among the most prevalent diseases affecting dairy cattle, primarily caused by bacterial infections that impact the udders of cows (*Bos taurus*) in both subtropical [1] and tropical regions [2]. These bacterial infections compromise the immune system, resulting in decreased milk yield and diminished milk quality [2]. The associated inflammatory response involves the mobilization and retention of hematopoietic cells at the site of infection to initiate tissue repair. Intramammary infection leads to damage to the ductal and secretory epithelium, thereby creating pathways between secretory cells and increasing the permeability of blood capillaries [3].

Microbial pathogens are detected by the innate immune system, which initiates immediate defense mechanisms and facilitates the development of long-lasting adaptive immunity [4]. This recognition system identifies the location, viability, replication, and pathogenic potential of microbial invaders [5]. The primary causative agents of mastitis are typically Gram-positive bacteria, including *Staphylococcus* spp., *Streptococcus* spp., and *Bacillus* spp. [6]. Interactions between pathogen-associated ligands and host cell wall components activate signaling cascades mediated by toll-like receptors (TLRs), which are essential for initiating the immune response.

The TLR family belongs to a group of pattern recognition receptors (PRRs) that play a distinctive role in detecting the onset of infection and are potent inducers of inflammatory responses [7]. This specificity is due to the combinatorial coding inherent to TLRs, with TLR1–10 identified in most mammals and TLR11–13 present in mice. Fisher *et al*. [8] emphasized that TLRs constitute the first line of defense, serving as innate immune sensors that monitor and identify a wide range of pathogenic challenges. TLRs act as critical barriers against pathogenic microorganisms.

TLR2 is expressed on the cell surface and binds to peptidoglycan and lipoteichoic acid (LTA) components of Gram-positive bacteria, thereby activating downstream signaling that leads to nuclear factor-κB (NF-κB) activation and subsequent production of proinflammatory cytokines [9]. Both TLR2 and TLR4 signal through the MyD88-dependent pathway to recruit receptor-associated genes [10], ultimately activating kinases that regulate transcription factors responsible for proinflammatory gene expression. Concurrent activation of these receptors also triggers the IκB kinase (IKK) family, leading to the activation of the NF-κB pathway through the IκBα inhibitor [11]. This activation enhances the transcription of inflammatory cytokine genes, including interleukin (IL)-6 and IL-1 alpha (IL-1α). Moreover, NF-κB signaling promotes the expression of chemotactic genes that mobilize neutrophils, dendritic cells (DCs), and natural killer cells, including IL-8 and the chemokine receptor gene (CXCR1) [12].

CXCR1, a member of the CXCRs family, plays a critical role in restraining bacterial infections by promoting neutrophil activity [13]. Mammary epithelial cells (MECs) are derived from mammary stem cells, which differentiate into epithelial precursor cells, giving rise to alveolar progenitors and lacteal duct cells [14]. Estrogen and progesterone promote the development of ductal precursors into basal and luminal epithelial cells, with alveolar cells proliferating substantially during pregnancy [15]. During lactation, these alveolar cells differentiate into secretory cells responsible for milk production. Utilizing epithelial cells to study immune responses against pathogenic bacterial exposure provides valuable insights into host-pathogen interactions [15].

Cell lines are derived from primary cultures and maintained continuously in *in vitro* conditions using media supplemented with low serum concentrations (approximately 2%) [16]. The HC11 cell line, originating from mid-pregnancy BALB/c mice, comprises mammary stem or progenitor cells [17].

Genetic resistance strategies have the potential to reduce the prevalence of diseases that compromise innate immunity. These approaches enable selective proliferation of cells capable of mounting effective and protective innate immune responses [18]. However, identifying genetic contributions to mastitis resistance remains challenging due to the complexity of host-pathogen interactions [18].

Although mastitis is a widely studied disease in dairy cattle, research has predominantly focused on major pathogens such as *Staphylococcus aureus* and *Escherichia coli*, with limited emphasis on Gram-positive *Bacillus* species. Moreover, the molecular mechanisms by which *Bacillus cereus* and *Bacillus subtilis* interact with MECs, particularly through PRRs such as TLRs and CXCRs like CXCR1, remain poorly understood. Most existing studies rely on *in vivo* models, where the complexity of systemic immune responses may obscure cell-specific signaling events. There is a critical need for *in vitro* investigations that isolate epithelial cell responses to specific pathogens. To date, no comparative analysis has comprehensively characterized the temporal gene expression patterns of TLR and CXCR family members in response to *Bacillus*-induced subclinical mastitis using the HC11 murine MECs model. This represents a significant gap in the current understanding of host-pathogen interactions at the epithelial interface.

The aim of this study was to investigate the differential expression of proinflammatory resistance genes - specifically TLR2, TLR4, IL-6, IL-1α, IL-8, and CXCR1 - in mammary epithelial HC11 cells challenged with *B. cereus* and *B. subtilis*. By employing a high-resolution time-course analysis over a 72-h period post-infection, we sought to elucidate the dynamics of innate immune signaling pathways and identify potential gene markers indicative of mastitis resistance or susceptibility. This research provides novel insights into the molecular basis of epithelial immune responses to Gram-positive mastitis pathogens and may contribute to the development of diagnostic and therapeutic strategies for improving udder health in dairy cattle.

## MATERIALS AND METHODS

### Ethical approval

Ethical approval was not necessary for this study. The samples were collected in accordance with standard collection procedure without any harm or unnecessary discomfort to the animals.

### Study period and location

The study was conducted from September 2016 to August 2017 in the Animal Health Laboratory, Biotechnology, Indonesian Institute of Sciences (LIPI).

### Cell lines

Expression studies and profiling of mastitis resistance genes, including members of the *TLR* and *CXCR* gene families, in response to bacterial infections associated with subclinical mastitis in dairy cow milk, were conducted using mammary epithelial HC11 cell cultures. The HC11 cells were provided by the Friedrich Miescher Institute, Switzerland, and the Department of Community Sciences, Bogor Agricultural University. The proliferation medium for HC11 cells consisted of RPMI 1640 supplemented with 10% fetal bovine serum, gentamicin (50 µg/mL), insulin (5 µg/mL), and epidermal growth factor (10 ng/mL).

### Sample collection

**Figure 1:**
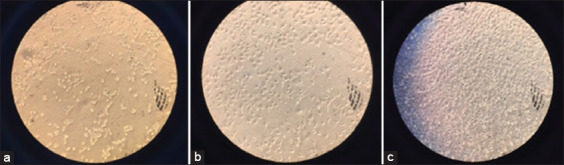
The proliferation of HC11 cell cultures: (a) culture conditions of HC11 cells at an initial concentration of 10^5^/mL cells, (b) culture conditions of HC11 cells at 40% of the initial concentration, and (c) confluent condition 80%–90% of the initial concentration of cell culture.

HC11 cells (10^5^ cells/mL) were incubated in a sterile environment containing 95% O_2_ and 5% CO_2_ at 36°C–37°C for 48 h until reaching confluence (Figures 1a and b). Following this, the medium was replaced with antibiotic-free RPMI 1640, and the cells were challenged with *B. cereus* and *B. subtilis* at a concentration of 5 × 10^7^ colony-forming units/mL [19]. This study employed an innovative time-course design, monitoring gene expression changes at multiple interval (0, 6, 12, 24, 48, and 72 h) post-infection. By integrating a high-resolution temporal framework, the study provides novel insights into the dynamic regulation of HC11 cells in response to *Bacillus*-induced mastitis. The incubation period required to achieve 80% confluence in the proliferation medium was 48 h. At each time point, cells were collected, centrifuged, and subjected to RNA extraction using the RNeasy® Mini Kit (Qiagen, Germany), following the manufacturer’s protocol. Gene expression was quantified using real-time polymerase chain reaction (PCR) with the SensiFAST™ SYBR No-ROX One-Step Kit (Bioland, USA).

### PCR analysis

Detection of *TLR* and *CXCR* gene expression was performed using the Illumina Eco real-time PCR system in accordance with the Bioland program protocol. The thermal cycling protocol included reverse transcription at 45°C for 10 min, polymerase activation at 95°C for 2 min, followed by 40 cycles of denaturation at 95°C for 5 s and annealing at 58°C for 10 s (optimized for target gene melting temperature), and a melt curve stage at 95°C for 15 s. Each reaction was performed in duplicate, and the mean cycle threshold (Ct) value was used to quantify gene expression. Normalization of target gene expression was performed using the mean Ct value of the housekeeping gene glyceraldehyde-3-phosphate dehydrogenase (GAPDH). The primer sequences used for six target genes (IL-6, IL-8, TLR2, IL-1α, TLR4, and CXCR1), along with GAPDH, are listed in Table 1. All primers were designed using the Primer3Plus software.

**Table 1:** Base sequences of primers for amplifying mastitis resistance genes.

No.	Gene	Primer	Base sequences of primers	Length (bp)
1.	*TLR2*	Reverse	5′-GAC CTG AAC CAG GAG GAT GA-3′	199
		Forward	5′-GCC TCG ACC TGT CCA ACA AT-3′	
2.	*TLR4*	Reverse	5′-TTA CGG CTT TTG TGG AAA CC-3′	213
		Forward	5′-TGC TGG CTG CAA AAA GTA TG-3′	
3.	*IL-1A*	Reverse	5′-GAG TCG GAC ATG ACT GAG CA-3′	189
		Forward	5′-GTC TCA GTT TGC TGCTGC TG-3′	
4.	*IL-6*	Reverse	5’-TAA GTT GTG TGC CCA GTG GA-3′	182
		Forward	5′-TGC AGT CTT CAA ACG AGT GG-3′	
5.	*IL-8*	Reverse	5′-CAG ACC TCG TTT CCA TTG GT-3′	190
		Forward	5′-TGC TCT CTG CAG CTC TGT GT-3′	
6.	*CXCR1*	Reverse	5′-GAC CTA GAT GAG GGG GTT GA-3′	191
		Forward	5′-TCA TCT TTG CTG TCG TGC TC-3′	
7.	*GAPDH*	Reverse	5′-TGG AAA CAT GTG GAA GTC AGG-3′	130
		Forward	5′-GGC CTC CAA GGA GTA AGG T-3′	

*TLR2*=Toll-like receptors 2, *IL*=Interleukin, *CXCR1*=Chemokine receptors 1, *GAPDH*=Glyceraldehyde-3-phosphate dehydrogenase

### Statistical analysis

The condition of HC11 cell proliferation in RPMI 1640 culture medium was assessed before and after bacterial challenge. Gene expression was analyzed through real-time PCR to determine the presence and quantity of resistance genes in RNA samples. Data were statistically evaluated using the Spearman correlation method to assess relationships between gene expression variables and the subclinical mastitis condition. The delta Ct (ΔCt) value was calculated by subtracting the Ct value of the housekeeping gene from the Ct value of the target gene (ΔCt = Ct_target − Ct_GAPDH) [20].

## RESULTS

**Figure 2:**
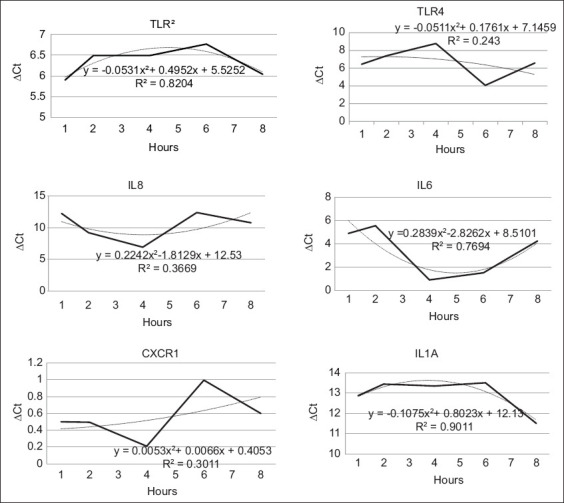
Immune gene expression was observed at time intervals in *Bacillus cereus*.

**Figure 3:**
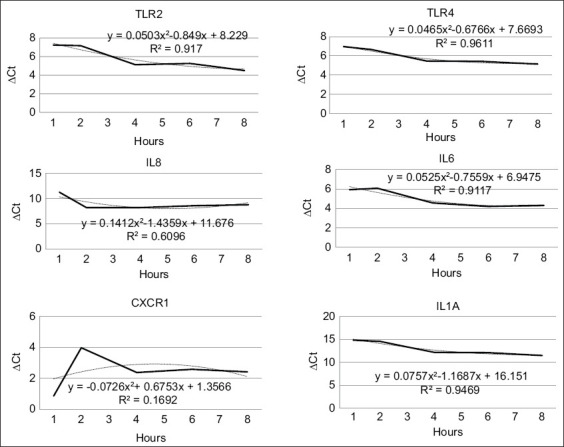
Immune gene expression was observed at time intervals in *Bacillus subtilis* bacteria.

The expression patterns of immune-related genes, particularly TLRs and CXCRs, in MECs were evaluated over a 72-h period following bacterial infection, as illustrated in Figures 2 and 3. At 0 h, Abbas *et al*. [21] noted that immune defense mechanisms in mast cells begin to activate. Observations at this initial time point revealed elevated expression levels across nearly all studied genes for both bacterial species. According to Piliponsky *et al*. [22], mast cells, which function as progenitor immune cells, are widely distributed throughout various tissues, capable of environmental sensing, and act as sentinel cells that respond to infection. The activation of immune gene expression was observed to commence at 6 h post-infection, as shown in Figure 2 and in the 3-time interval method.

Following 6 h of bacterial challenge, cellular responses indicated gene-specific activation dependent on ligand-pathogen interactions. Previous research by Yanthi [23] identified Gram-positive bacteria as the predominant cause of subclinical mastitis in West Java. Particular attention was given to LTA, a glycolipid present in the membranes of Gram-positive bacteria. LTA, comprising an acylated glycerol backbone linked to diverse carbohydrate moieties through ether bonds, is a ligand recognized by TLR2. The high correlation coefficient (r^2^) values between both bacterial treatments suggest that gene expression during infection is closely associated with these molecular interactions. TLR2, a transmembrane receptor, plays a vital role in recognizing specific ligands derived from Gram-positive bacteria. A novel finding of this study was the biphasic expression pattern of TLR2 – characterized by early upregulation followed by suppression – possibly indicating receptor desensitization or immune feedback regulation. This challenges the conventional understanding that TLR activation follows a linear, sustained trajectory.

Alongside TLR2, the *TLR4* gene is also integral in initiating immune responses against a wide range of pathogens, including both Gram-negative and Gram-positive bacteria, as well as fungi and viruses [24]. TLR4 expression patterns revealed notable differences between the two bacterial species: *B. cereus* exhibited a markedly lower r^2^ value, whereas *B. subtilis* showed a significantly higher r^2^ value.

The *IL-6* gene, encoding a pleiotropic cytokine, is expressed by various cell types, including stromal, hematopoietic, epithelial, and muscle cells [25]. In contrast to prior studies that predominantly associate IL-6 with acute inflammatory responses, our results demonstrated a sustained upregulation of IL-6 following *B. subtilis* infection, whereas a transient response was observed with *B. cereus*. These findings suggest that different mastitis-causing pathogens may employ distinct strategies to evade or modulate host immune responses.

IL-8, a chemokine, plays a central role in chemotaxis by directing leukocyte migration to sites of inflammation [26]. This chemotactic activity is evident in Figures 2 and 3, where the directional movement pattern of IL-8 reflects migration toward the inflammatory center. The recruitment of neutrophils from circulation into the mammary gland is essential for enhancing the gland’s immune response. IL-8 exerts its function by binding to CXCRs, particularly CXCR1 (CXC motif 1), which in turn initiates chemotaxis and strengthens mammary immune defense [27]. Furthermore, CXCR1 activation supports neutrophil survival through the induction of bovine genes responsive to live bacterial stimulation and early inflammatory mediators, including tumor necrosis factor (TNF) and IL-1 [28–30]. Although the overall expression level of CXCR1 was lower than that of other immune genes, its expression consistently increased from the 0-h mark through to 72 h post-infection.

IL-1α is a biologically active cytokine expressed in numerous cell types. It does not require further processing upon activation, unlike some other cytokines. Both IL-1α and IL-1β, members of the IL-1 family, are key mediators involved in inducing fever, facilitating infection response, activating neutrophils, recruiting monocytes, synthesizing prostaglandins, and stimulating T and B cell responses as well as cytokine production [31]. The consistent presence of IL-1α across cell types contributed to its robust expression throughout the 0–72 h period (Figure 3), with distinct response patterns observed for each bacterial treatment.

## DISCUSSION

Innate immunity encompasses a complex network of mechanisms involving leukocytes and their interactions with various physiological systems to confer resistance against microbial pathogens. This integrated system collectively protects the host from invading organisms [32]. Host defense operates through two primary strategies: resistance, which involves the elimination of pathogens or harmful stimuli, and tolerance, which refers to the capacity of cells to minimize tissue damage without necessarily reducing pathogen viability [33].

The coordinated activation of immune genes by various cell types plays a vital role in maintaining microbial homeostasis in infected tissues. The udder comprises diverse cell populations, each exhibiting unique immune characteristics determined by their level of immune competence [34]. The interaction among these cells is essential in orchestrating the immune response during the early stages of udder infection [3]. The HC11 MECs line, composed of epithelial cells, is crucial for detecting bacterial invasion [35]. These cells express PRRs that recognize pathogen-associated molecular patterns (PAMPs), serving as the first line of defense against intramammary infection [36]. This study enhances current understanding by demonstrating that HC11 cells exhibit gene-specific immune modulation depending on the bacterial species involved. The results suggest that host responses to Gram-positive bacteria are not uniform but rather tailored to the specific virulence factors of each pathogen. The observed variation in gene expression reflects differences in the host’s immunological engagement with *B. cereus* and *B. subtilis*.

Both bacterial species triggered similar immune activation profiles, notably increasing the expression of TLR4 and IL-6. This upregulation is likely due to the activation of TLR2-associated defense ligands in response to Gram-positive bacteria [24], which in turn signal epithelial cells and macrophages to elevate the expression of additional immune-related genes as part of the tolerance response. Conversely, TLR2 expression was downregulated following infection, potentially indicating an evolved resistance mechanism to prevent tissue infiltration by Gram-positive pathogens. IL-6 expression was upregulated in response to both bacterial species [37], consistent with findings from Kawecka-Grochocka *et al*. [38], who reported IL-6 induction in MECs upon Gram-positive bacterial challenge. The *IL-1α* gene, another key proinflammatory cytokine, also exhibited increased expression.

According to Wu *et al*. [28], IL-8 levels are elevated in both acute and chronic phases of subclinical mastitis, including during the dry period. Consistent with our results, IL-8 levels increased post-infection. Neutrophils play a central role in innate defense by phagocytosing bacteria and releasing reactive oxygen species and antimicrobial factors [39]. During mastitis, neutrophils become the predominant leukocyte population in the mammary gland, surpassing macrophages [3].

*TLR* genes are critical components of the host defense system, mediating responses through the TLR signaling pathway [40]. These receptors recognize microbial ligands embedded in the cell wall, activating downstream signaling to coordinate epithelial cell activity at the infection site. The mammary gland’s immune response involves a concerted action between surface-bound and inducible genes, including proinflammatory cytokines that orchestrate pathogen defense [41].

Key proinflammatory cytokines – TNFα, IL-1, IL-6, and interferon-gamma – stimulate the production of additional cytokines and chemokines that bind to receptors on epithelial cells [42, 43]. TLR activation also induces inflammatory cytokine expression, initiates immune responses, regulates cell differentiation, and promotes apoptosis [44, 45].

This study conducted gene expression analysis on HC11 cells exposed to mastitis-causing bacteria found in milk. Different bacterial species contribute variably to mammary tissue damage [46]. Gram-negative bacteria typically induce rapid inflammatory responses leading to clinical mastitis, whereas Gram-positive bacteria often provoke more prolonged, subclinical infections that may progress to chronic inflammation and prove more challenging to treat [46]. Controlling Gram-positive pathogens, which are prevalent in dairy environments, remains a significant concern.

Inflammation is a protective immune response to harmful stimuli, such as pathogens, dead cells, and irritants. Subclinical inflammation, in particular, can lead to chronic systemic disorders [47]. The innate immune system uses PRRs to detect both PAMPs and danger-associated molecular patterns (DAMPs), which signal cellular stress. PRR activation triggers downstream signaling cascades that release type I interferons (interferon-α and interferon-β) and proinflammatory cytokines [47].

Sterile inflammation, initiated by DAMPs in the absence of pathogens, is also a feature of chronic inflammatory diseases [48]. Following PAMP recognition, MECs rapidly signal for the recruitment of immune effector cells, such as neutrophils and macrophages, to the infection site [38]. Neutrophil mobilization from the bloodstream can significantly alter tissue dynamics within 24 h, enhancing pathogen resistance [49].

Macrophages act as early responders to infection by producing proinflammatory cytokines. This defense response is initiated when host receptors detect microbial patterns, activating pathways that upregulate these cytokines [50].

The TLR pathway is essential in recognizing PAMPs. In Gram-positive bacteria, LTA serves as a TLR2 ligand, initiating NF-κB activation and inflammatory signaling cascades [51]. NF-κB transcription factors are pivotal regulators of immune and inflammatory responses [52]. TLR2 and TLR4 exhibit strong co-expression, suggesting coordinated regulation during udder infection [53].

Interestingly, *B. cereus* and *B. subtilis* both induced significant increases in TLR4 mRNA in HC11 cells [54, 55], despite previous findings by Chantratita *et al*. [56] suggesting that Gram-positive bacteria do not directly activate TLR4. Instead, TLR4 appears to modulate inflammatory responses to Gram-positive pathogens, reflecting complex crosstalk within the immune network.

*In situ* hybridization revealed high expression of TLRs in MECs from infected glands, underscoring their role as pathogen sensors [57]. These receptors are predominantly expressed by antigen-presenting cells, such as macrophages and DCs, and are activated by PAMPs [58], playing crucial roles in both innate and adaptive immunity.

IL-1 is a proinflammatory cytokine family comprising IL-1α, IL-1β, and IL-1 receptor antagonists. These molecules regulate inflammation by inducing vasodilation and promoting leukocyte migration [29, 59, 60]. While they amplify immune responses, their overactivation can lead to tissue damage, particularly when mediated by caspase-1-dependent pathways [61].

Proteins encoded by these genes belong to the IL-1 family and are involved in inflammation, immune responses, and hematopoiesis. Monocytes and macrophages typically synthesize them as proproteins, which are cleaved and activated in response to cell injury, often inducing apoptosis [62].

The inflammasome – a multiprotein complex assembled in response to PAMPs or DAMPs – is a central component of innate immunity [63]. Beyond IL-1, other early-response cytokines, such as IL-8, also contribute to antimicrobial defense in MECs [64]. IL-8, produced by epithelial and endothelial cells, among others, facilitates phagocytosis and chemotaxis at infection sites [29, 60].

*IL-8* gene expression was significantly upregulated in early infection phases, consistent with Wu *et al*. [28]. MECs play a vital role in neutrophil recruitment and activation during early post-infection stages through the IL-8 signaling axis [49].

IL-8 acts as a strong chemoattractant for neutrophils and other granulocytes during mastitis [59]. Cabrini *et al*. [65] highlighted IL-8’s essential role in neutrophil recruitment as a defining feature of epithelial immune responses. The strong chemokine induction in epithelial cells supports their role in guiding polymorphonuclear neutrophils to the mammary gland to combat infection [66]. A lack of sufficient cytokine signaling reduces chemokine production and impairs neutrophil chemotaxis, heightening susceptibility to infection [65].

Observations following *B. subtilis* infection indicated increased IL-6 expression after 6 h. IL-6, a pleiotropic cytokine secreted by monocytes and macrophages, is pivotal in vascular inflammation and infection defense. Its expression varies based on pathogen type and immune context. Epigenetic factors may also modulate IL-6 expression in mammary cells [42]. Wu *et al*. [28] reported that abnormal physiological conditions can suppress proinflammatory gene expression, diminishing immune responsiveness.

A notable finding in this study was the delayed but sustained expression of CXCR1, indicating a secondary phase of immune activation linked to prolonged neutrophil recruitment. This diverges from the previously assumed concurrent expression of CXCR1 with IL-8. According to Matsushima *et al*. [29], CXCR1, expressed on neutrophils, binds to CXCL8 (IL-8), facilitating neutrophil chemotaxis. Goulart and Mellata [59] also noted that neutrophil recruitment is a key defense mechanism during mastitis. In this study, IL-8 appeared to exert a more dominant influence than CXCR1. CXCR1 expression notably increased at 12 h post-infection. However, its overall expression remained lower than other resistance genes in HC11 cells exposed to intramammary infection.

Infections were experimentally induced in HC11 cells using *B. cereus* and *B. subtilis*, two prominent mastitis-associated bacteria previously underreported in gene expression studies. Notably, the expression of immune genes was higher in response to *B. cereus* compared to *B. subtilis*.

The host response to Gram-positive infections in HC11 cells is mediated through the MyD88-dependent pathway, activated by TLRs. Expression of *TLR* genes was promptly induced at 0 h post-infection [68].

According to Kawecka-Grochocka *et al*. [38], TLR2 on epithelial cell surfaces binds peptidoglycan and LTA from Gram-positive bacteria, initiating a signaling cascade that activates NF-κB and stimulates cytokine production. Both TLR2 and TLR4 utilize the MyD88-dependent signaling axis.

This pathway recruits genes, such as IRAK4, IRAK1, TRAF6, TAB1, TAB2, and TAK1 [69], which activate transcription factors that induce proinflammatory cytokines. This signaling cascade also activates the IKK complex, resulting in IκBα phosphorylation and subsequent NF-κB activation [70]. NF-κB regulates expression of IL-6 and IL-1A and other inflammatory genes [71], while also stimulating chemotactic genes such as IL-8 and CXCR1 [72]. CXCR1, part of the CXCRs family, is notably active in neutrophil-mediated bacterial defense [73].

## CONCLUSION

This study provides novel insights into the immunogenetic response of mammary epithelial HC11 cells to Gram-positive mastitis-causing bacteria, *B. cereus* and *B. subtilis*. Our time-course gene expression analysis demonstrated that immune gene activation in epithelial cells is both time-dependent and pathogen-specific. Notably, TLR2 exhibited a biphasic expression pattern with early upregulation followed by suppression, potentially reflecting receptor desensitization. In contrast, TLR4 showed higher expression in response to *B. subtilis*, suggesting differential signaling roles in pathogen recognition. Proinflammatory cytokines IL-6 and IL-1α were significantly upregulated post-infection, while IL-8 and CXCR1 were differentially expressed, indicating their involvement in neutrophil recruitment and immune modulation.

The strength of this study lies in its high-resolution temporal analysis and the use of a well-characterized MECs model (HC11), which enabled precise monitoring of host-pathogen interactions without systemic confounding factors. In addition, the comparative approach revealed distinct gene expression profiles between the two *Bacillus* species, challenging the assumption that all Gram-positive bacteria elicit similar immune responses.

However, this study is subject to certain limitations. The use of an *in vitro* monoculture model does not fully capture the complexity of immune cell interactions, hormonal influences, and systemic regulation present *in vivo*. Furthermore, the reliance on mRNA expression does not account for post-transcriptional and post-translational modifications that could influence protein activity and immune outcomes.

Future research should explore the downstream signaling pathways activated by TLR2 and TLR4 in response to different bacterial ligands, incorporating proteomic and functional assays to validate gene expression data. In addition, expanding this work to include co-culture models with immune cells or *in vivo* validation in dairy animals could provide a more holistic understanding of mastitis pathophysiology. The identification of IL-1α, IL-6, and CXCR1 as key modulators offers promising avenues for the development of molecular biomarkers and genetic selection strategies aimed at enhancing mastitis resistance in dairy herds.

## AUTHORS’ CONTRIBUTIONS

NDY and MM: Planned and designed the study. NDY: Sample collection. NDY, AA, SS, and RD: Conducted the study and analyzed the results. SuS, NH, IF, HAN, MFN, and RT: Research and media preparation. NDY and AA: Wrote the manuscript. AA and IF: Reviewed and edited the manuscript. All authors have read and approved the final manuscript.
